# Association between dietary index for gut microbiota and diarrhea among US adults: a cross-sectional analysis of NHANES 2005–2010

**DOI:** 10.3389/fnut.2025.1566314

**Published:** 2025-04-11

**Authors:** Xiaoqiang Liu, Xiaobo Liu, Yingxuan Huang, Chanchan Lin, Xinqi Chen, Yingyi Li, Yisen Huang, Yubin Wang

**Affiliations:** ^1^Department of Gastroenterology, First Hospital of Quanzhou Affiliated to Fujian Medical University, Quanzhou, China; ^2^McConnell Brain Imaging Centre, Montreal Neurological Institute, McGill University, Montreal, QC, Canada

**Keywords:** gut microbiota, diarrhea, dietary index for gut microbiota, cross-sectional study, NHANES

## Abstract

**Objectives:**

Growing attention has been paid to the relationship between the gut microbiota and diarrhea. A recently proposed Dietary Index for Gut Microbiota (DI-GM) reflects the overall dietary quality as it pertains to gut microbiota diversity. However, evidence regarding the association between DI-GM and diarrhea is still lacking. This study aims to investigate the association between DI-GM and the risk of diarrhea.

**Methods:**

A total of 15,590 adults (≥20 years old) from National Health and Nutrition Examination Survey (NHANES) 2005–2010 were included in this analysis. Diarrhea was defined by self-reported common Bristol Stool Form Scale (BSFS) type 6 or 7, or more than three bowel movements per day. DI-GM comprises 14 food/nutrient components known to be associated with gut microbiota. A higher score indicates a more favorable diet for the gut microbiota. Multivariable logistic regression was used to evaluate the association between DI-GM and diarrhea, with subgroup and sensitivity analyses performed to confirm robustness.

**Results:**

After adjusting for age, gender, race, physical activity, chronic diseases, and other confounders, each 1-point increase in DI-GM was associated with a 5% reduction in diarrhea risk (adjusted OR = 0.95, 95% CI: 0.91–0.98, *p* = 0.005). Compared with those who had a DI-GM score of 0–3, individuals with a DI-GM score ≥ 6 demonstrated a significantly lower risk of diarrhea (OR = 0.77, 95% CI: 0.65–0.91, *p* = 0.002). Subgroup and sensitivity analyses further supported this negative association. Notably, the “beneficial component” was found to have a more pronounced effect on reducing diarrhea risk.

**Conclusion:**

Based on a large representative population, our findings suggest that a higher DI-GM score is significantly associated with a lower risk of diarrhea, underscoring the importance of overall dietary patterns in maintaining gut function and homeostasis.

## 1 Introduction

Diarrhea is a common gastrointestinal symptom and a global public health concern, with etiologies ranging from infections and medications to metabolic abnormalities. According to the World Health Organization, diarrhea remains one of the leading causes of morbidity and mortality worldwide, particularly in low- and middle-income countries, although it also poses a significant clinical burden in developed regions (1). Epidemiological data indicate that up to 3–5% of adults in the general population report frequent diarrhea symptoms, and it is also frequently observed in functional bowel disorders such as irritable bowel syndrome (IBS) (2–4). Although multiple interventions exist to alleviate diarrhea, clinical outcomes and recurrence rates still vary considerably among individuals (5). With recent advances in microbiome research, the critical role of gut microbiota in intestinal homeostasis, immune modulation, and the onset and progression of diarrhea has gained increasing attention (6–8).

Existing studies have shown that diet is a key exogenous factor influencing gut microbiota composition and activity (9, 10). High-fiber diets, probiotics, and fermented dairy products can promote the proliferation of beneficial gut microbes and improve intestinal barrier function, thereby lowering the risk of diarrhea (11, 12). In contrast, excessive intake of red meat, refined sugars, and high-fat foods can disturb gut microbiota stability, increase intestinal permeability, and ultimately lead to inflammation or diarrhea (13, 14). Despite some success in interventions targeting single nutrients or specific foods, the multifaceted and complex relationship between dietary structure and the gut microbiota remains insufficiently quantified (15).

To address this gap, Kase et al. introduced a new dietary quality assessment metric in 2024—the Dietary Index for Gut Microbiota (DI-GM) (16). DI-GM integrates various foods and nutrients closely associated with gut microbiota health, encompassing beneficial items (e.g., high-fiber, fermented dairy, whole grains) and unfavorable components (e.g., high-fat, red meat, processed meats). This index aims to capture the potential impacts of overall dietary patterns on gut microbiota diversity and function. Some recent research based on the National Health and Nutrition Examination Survey (NHANES) population in the United States has linked higher DI-GM scores to lower risk of chronic diseases like diabetes and functional bowel disorders such as constipation (17, 18). However, whether a similar association exists between DI-GM and diarrhea remains unexplored.

Accordingly, using large-scale cross-sectional data from NHANES 2005–2010, the present study aimed to investigate, for the first time, the association between DI-GM and diarrhea. We hypothesized that, after adjusting for demographic characteristics, lifestyle factors, and other common chronic conditions, higher DI-GM scores would be associated with lower diarrhea risk. By analyzing a large, diverse population, this study seeks to provide insights that may inform clinical prevention and management strategies for diarrhea through dietary modulation of the gut microbiota.

## 2 Methods

### 2.1 Data source

This study was based on data from the 2005–2010 NHANES, a nationally representative cross-sectional survey of the non-institutionalized U.S. population. NHANES employs a complex, multistage probability sampling method to collect health, nutrition, and demographic data. All data used in this study are publicly available.^1^ Ethical approval was obtained from the National Center for Health Statistics Research Ethics Review Board, and all participants provided informed consent. The reporting of this study followed the Strengthening the Reporting of Observational Studies in Epidemiology (STROBE) guidelines (19).

### 2.2 Study design and participants

**Figure 1:**
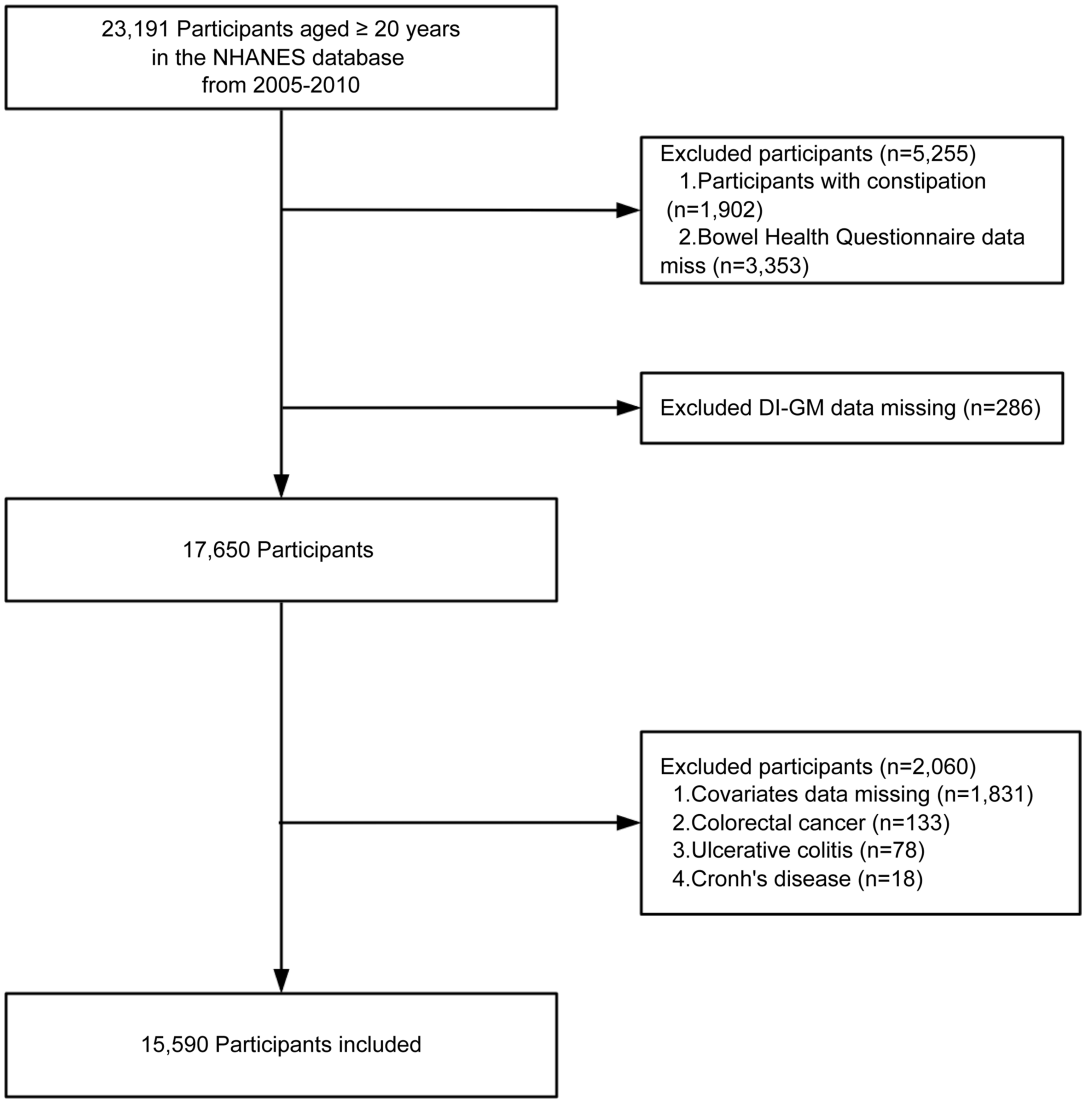
Flow chart of the screening of the NHANES 2005–2010 participants. DI-GM, dietary index for gut microbiota.

We included adults aged ≥20 years from NHANES 2005–2010. Exclusion criteria encompassed the presence of constipation, a confirmed diagnosis of colorectal cancer, ulcerative colitis, or Crohn’s disease, lack of a DI-GM score, absence of Bowel Health Questionnaire data, or insufficient essential covariate information. A total of 15,590 eligible participants were finally included in the analysis, of whom 1,498 were classified as having diarrhea and 14,092 as without diarrhea (Figure 1).

### 2.3 Definition of diarrhea

Diarrhea was defined based on responses to the Bowel Health Questionnaire administered via computer-assisted personal interviews (CAPI) in mobile examination centers (MEC). Participants were shown a color card illustrating the seven types of the Bristol Stool Form Scale (BSFS, Types 1–7) and were asked: “Please look at this card and tell me the number of the stool type that best fits your usual or common stool form.” Consistent with previous studies, chronic diarrhea was defined as self-reported usual stool types corresponding to BSFS Type 6 (fluffy pieces with ragged edges) or Type 7 (watery, no solid pieces). Chronic constipation was defined as BSFS Type 1 (separate hard lumps like nuts) or Type 2 (sausage-shaped but lumpy). Participants who did not meet these criteria were classified as having normal bowel habits (20, 21). To assess stool frequency, participants were asked, “How many times a week do you usually have a bowel movement?” In this study, individuals reporting two or fewer bowel movements per week were classified as having constipation, whereas diarrhea was defined as more than three bowel movements per day (22). Participants who had used laxatives within the past month were excluded. For the purposes of analysis, participants who met either the stool form based or stool frequency based criterion for diarrhea were classified as having diarrhea. Conversely, participants who did not meet either the stool form based or stool frequency based criteria for diarrhea or constipation were classified as having normal bowel habits.

### 2.4 Assessment of the dietary index for gut microbiota

The Dietary Index for DI-GM is a novel diet quality index developed to capture the potential impact of dietary components on gut microbiota diversity (16). It comprises 14 foods and nutrients theoretically linked to gut microbiota health, including 10 “beneficial” components (avocado, broccoli, chickpeas, coffee, cranberries, fermented dairy products, fiber, green tea, soy, and whole grains) and 4 “unfavorable” components (red meat, processed meat, refined grains, and a high-fat diet, defined as ≥40% of total energy from fat). However, because NHANES does not differentiate green tea from other teas, that component could not be scored in our analyses. As a result, we ultimately used 13 components to derive the total DI-GM score.

Each food/nutrient is scored based on sex-specific median intake. Participants receive 1 point if intake of beneficial components is above the median (or if fat intake is below 40% for the high-fat diet component), and 0 points otherwise. Conversely, participants receive 1 point if unfavorable components are below the median, and 0 points otherwise. The components along with scoring criteria for the DI-GM can be found in Supplementary Table 1. Total DI-GM scores range from 0 to 13. Higher scores indicate a diet more likely to benefit the gut microbiota. Based on prior research and a quartile-based approach, DI-GM scores were categorized into four groups: 0–3, 4, 5, and ≥6 (23).

### 2.5 Covariates

Based on previous studies and clinical judgment (20, 24), we adjusted for a range of potential confounders, including age, gender, race, marital status, education level, poverty-to-income ratio (PIR), physical activity, smoking status, alcohol use, body mass index (BMI), total energy intake, diabetes, cardiovascular disease (CVD), hypertension, hyperlipidemia, and depression. Age was treated as a continuous variable. Gender referred to biological sex. Race was categorized as non-Hispanic White and “Other” (non-Hispanic Black, Mexican American, other Hispanic, and other races). Marital status was classified as married/living with partner versus unmarried/other (including widowed, divorced, or separated). Education level was categorized as <High school, High school/equivalent, and >High school. PIR was grouped as 1–1.3, 1.31–3.50, and >3.50 (25). Physical activity was categorized based on the time (MET-min/week) spent walking, bicycling, working, and recreational activities: inactive (0 MET-min/week), insufficiently active (1–599 MET-min/week), and sufficiently active (≥600 MET-min/week) (26). Smoking status was defined as never smokers (<100 cigarettes in lifetime), former smokers (≥100 cigarettes in lifetime but currently not smoking), or current smokers (≥100 cigarettes in lifetime and currently smoking) (27). Alcohol intake was categorized as never (lifetime <12 drinks), former (≥12 drinks in a given year but none in the past year, or no drinking in the past year but ≥12 drinks in a lifetime), or current drinkers (≥12 drinks in any year and drank in the past year) (28). BMI was calculated as weight (kg) divided by height squared (m^2^). Total energy intake was determined using the first 24 h dietary recall data (DR1TOT) from the NHANES. NHANES uses the Automated Multiple Pass Method (AMPM), an interviewer administered 24 h recall procedure that systematically captures all foods and beverages consumed from midnight to midnight the previous day. The reported foods are then coded, and total nutrient intakes, including kilocalories, are calculated, with DR1TOT representing total energy intake. Diabetes was defined by laboratory tests (HbA1c ≥ 6.5%, fasting plasma glucose ≥7.0 mmol/L, random/2-h OGTT glucose ≥11.1 mmol/L) and/or physician diagnosis or use of antidiabetic medication/insulin (29). CVD was based on self-reported physician diagnoses of coronary heart disease, angina, stroke, heart attack, or congestive heart failure. Hypertension was defined by systolic BP ≥140 mmHg and/or diastolic BP ≥90 mmHg, self-reported physician diagnosis, or use of antihypertensive medication (27). Hyperlipidemia was identified if any of the following criteria were met: use of lipid-lowering drugs, high triglycerides (≥150 mg/dL), or high cholesterol (total cholesterol ≥200 mg/dL, LDL ≥130 mg/dL, or HDL <40 mg/dL) (30). Depression was screened via the PHQ-9 questionnaire. A PHQ-9 score ≥ 10 was defined as depression (31).

### 2.6 Statistical analysis

All statistical analyses were performed using R (version 4.2.3) and Free Statistics software (version 1.9.2). Statistical significance was defined as a two-sided *P*-value < 0.05. Continuous variables were expressed as mean ± standard deviation (SD) for normally distributed data or as median (interquartile range) for skewed data. Categorical variables were described as counts (percentages). Between-group differences were compared using the independent samples t-test, chi-square test, and Mann–Whitney U test, as appropriate.

To assess the association between DI-GM and diarrhea, we constructed multivariable logistic regression models to estimate odds ratios (OR) and 95% confidence intervals (CI). The covariates included in the model were determined based on clinical expertise, univariate screening results (*P*-value < 0.05), or whether they led to a change in the effect estimate >10%. Model 1 adjusted for age and gender; Model 2 further included race, marital status, education level, PIR, physical activity, smoking status, alcohol intake, BMI, total calories intake, diabetes, CVD, hypertension, hyperlipidemia, and depression. We also categorized DI-GM into four groups (0–3, 4, 5, ≥6) to examine potential dose–response relationships. Restricted cubic spline (RCS) analyses with three knots (at the 10th, 50th, and 90th percentiles of DI-GM) were used to explore possible nonlinear associations. Subgroup analyses were conducted by stratifying participants according to age, gender, race, CVD, hypertension, hyperlipidemia, diabetes, and depression status, to test consistency across different population subgroups. For sensitivity analysis, multiple imputation by chained equations was used to handle missing values. Five imputed datasets were generated, and results were combined using Rubin’s rules (32).

## 3 Results

### 3.1 Baseline characteristics

Table 1 presents the basic characteristics of the study population. Among the 15,590 participants, 1,498 had diarrhea and 14,092 did not. The average age in the diarrhea group was 52.8 years (SD 16.4), which was significantly higher than the 49.4 years (SD 17.7) in the without diarrhea group (*p* < 0.001). The proportion of females was also higher in the diarrhea group than in the without diarrhea group (56.1% vs. 43.9%, *p* < 0.001). In addition, the diarrhea group had higher proportions of lower income (PIR ≤ 1.3), and lower education levels (all *p* < 0.001). The mean BMI in the diarrhea group was higher compared to the without diarrhea group (30.8 vs. 29.1 kg/m^2^, *p* < 0.001). Regarding diet, both total energy intake (2058.3 kcal vs. 2147.9 kcal, *p* = 0.001) and DI-GM scores (4.4 vs. 4.5, *p* < 0.001) were significantly lower in the diarrhea group compared to the without diarrhea group.

**Table 1:** Characteristics of the NHANES 2005–2010 participants.

Variables	Total	Without diarrhea	Diarrhea	Significance tests
Number of participants	15,590	14,092	1,498	
Age, Mean ± SD, year	49.7 ± 17.6	49.4 ± 17.7	52.8 ± 16.4	T, *P* < 0.001
Gender, *n* (%)				χ ^2^, *P* < 0.001
Male	8,014 (51.4)	7,356 (52.2)	658 (43.9)	
Female	7,576 (48.6)	6,736 (47.8)	840 (56.1)	
Race, *n* (%)				χ ^2^, *P* < 0.001
Non-Hispanic White	8,329 (51.5)	7,716 (52.2)	613 (44.9)	
Others	7,829 (48.5)	7,076 (47.8)	753 (55.1)	
Marital status, *n* (%)				χ ^2^, *P* = 0.501
Married/living with partner	9,596 (61.6)	8,686 (61.6)	910 (60.7)	
Never married/other	5,994 (38.4)	5,406 (38.4)	588 (39.3)	
PIR group, *n* (%)				χ ^2^, *P* < 0.001
1–1.3	4,519 (29.0)	3,953 (28.1)	566 (37.8)	
1.31–3.50	5,977 (38.3)	5,427 (38.5)	550 (36.7)	
>3.50	5,094 (32.7)	4,712 (33.4)	382 (25.5)	
Education level, *n* (%)				χ ^2^, *P* < 0.001
Less than high school	4,087 (26.2)	3,509 (24.9)	578 (38.6)	
High school or equivalent	3,660 (23.5)	3,311 (23.5)	349 (23.3)	
Above high school	7,843 (50.3)	7,272 (51.6)	571 (38.1)	
Smoking status, *n* (%)				χ ^2^, *P* = 0.098
Never	8,071 (51.8)	7,332 (52.0)	739 (49.3)	
Former	4,048 (26.0)	3,650 (25.9)	398 (26.6)	
Current	3,471 (22.3)	3,110 (22.1)	361 (24.1)	
Alcohol intake, *n* (%)				χ ^2^, *P* < 0.001
Never	1,851 (11.9)	1,636 (11.6)	215 (14.4)	
Former	2,962 (19.0)	2,620 (18.6)	342 (22.8)	
Current	10,777 (69.1)	9,836 (69.8)	941 (62.8)	
Physical activity, *n* (%)				χ ^2^, *P* < 0.001
Inactive	3,943 (25.3)	3,475 (24.7)	468 (31.2)	
Insufficiently active	3,148 (20.2)	2,845 (20.2)	303 (20.2)	
Sufficiently active	8,499 (54.5)	7,772 (55.2)	727 (48.5)	
BMI, Mean ± SD, Kg/m^2^	29.2 ± 6.8	29.1 ± 6.7	30.8 ± 7.8	T, *P* < 0.001
Diabetes, *n* (%)				χ ^2^, *P* < 0.001
No	12,837 (82.3)	11,701 (83.0)	1,136 (75.8)	
Yes	2,753 (17.7)	2,391 (17.0)	362 (24.2)	
CVD, *n* (%)				χ ^2^, *P* < 0.001
No	13,901 (89.2)	12,608 (89.5)	1,293 (86.3)	
Yes	1,689 (10.8)	1,484 (10.5)	205 (13.7)	
Hypertension, *n* (%)				χ ^2^, *P* < 0.001
No	9,007 (57.8)	8,266 (58.7)	741 (49.5)	
Yes	6,583 (42.2)	5,826 (41.3)	757 (50.5)	
Hyperlipidemia, *n* (%)				χ ^2^, *P* < 0.001
No	4,291 (27.5)	3,972 (28.2)	319 (21.3)	
Yes	11,299 (72.5)	10,120 (71.8)	1,179 (78.7)	
Depression, *n* (%)				χ ^2^, *P* < 0.001
No	14,307 (91.8)	13,083 (92.8)	1,224 (81.7)	
Yes	1,283 (8.2)	1,009 (7.2)	274 (18.3)	
Total calories intake, Mean ± SD, kcal	2139.3 ± 1011.3	2147.9 ± 1015.7	2058.3 ± 965.7	T, *P* = 0.001
DI-GM score, Mean ± SD	4.5 ± 1.5	4.5 ± 1.5	4.4 ± 1.5	T, *P* < 0.001
DI-GM group, *n* (%)				χ ^2^, *P* < 0.001
0–3	3,946 (25.3)	3,545 (25.2)	401 (26.8)	
4	3,953 (25.4)	3,528 (25.0)	425 (28.4)	
5	3,719 (23.9)	3,350 (23.8)	369 (24.6)	
≥6	3,972 (25.5)	3,669 (26.0)	303 (20.2)	
Beneficial to gut microbiota	2.0 (1.0, 3.0)	2.0 (1.0, 3.0)	2.0 (1.0, 3.0)	U, *P* < 0.001
Unfavorable to gut microbiota	2.3 ± 1.0	2.3 ± 1.0	2.3 ± 1.0	T, *P* = 0.973

DI-GM, dietary index for gut microbiota; BMI, body mass index; PIR, poverty income ratio; CVD, cardiovascular disease; χ ^2^, chi-square test; T, T-test; U, Mann–Whitney U test.

### 3.2 Association between DI-GM and diarrhea

**Figure 2:**
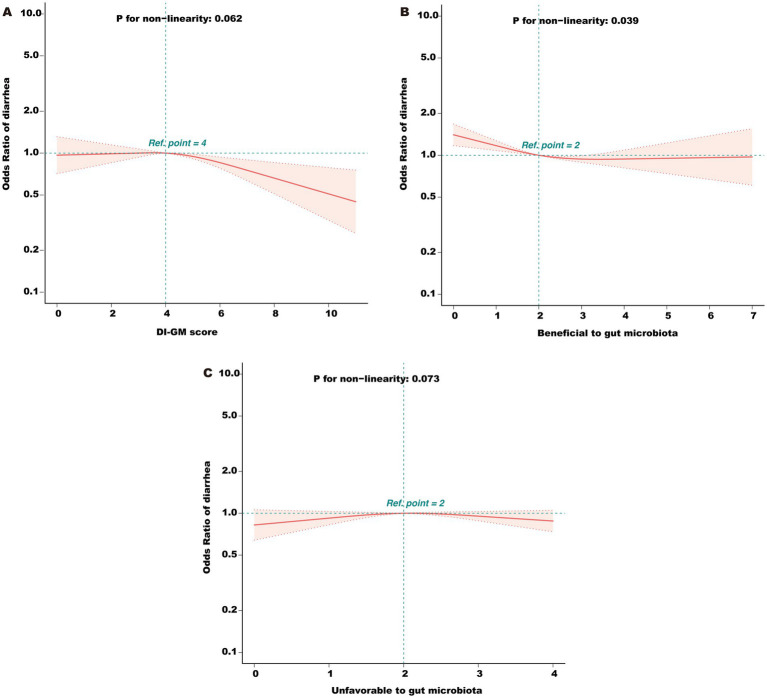
**(A)** Association between total DI-GM score and odds ratio (OR) of diarrhea. **(B)** Association between the beneficial component score and OR of diarrhea. **(C)** Association between the unfavorable component score and OR of diarrhea. DI-GM, dietary index for gut microbiota; PIR, poverty income ratio; BMI, body mass index; CVD, cardiovascular disease; RCS, restricted cubic spline. The model was adjusted for age, gender, race, marital status, education level, PIR, physical activity, smoking status, alcohol intake, BMI, total calories intake, diabetes, CVD, hypertension, hyperlipidemia, and depression.

Multivariable logistic regression analysis (Table 2) demonstrated that each 1-point increase in DI-GM was associated with a 5% reduction in diarrhea risk (adjusted OR = 0.95, 95% CI: 0.91–0.98, *p* = 0.005) after full covariate adjustment. Further, subgroup analyses by DI-GM categories showed that individuals with DI-GM ≥6 had a markedly lower diarrhea risk compared with those scoring 0–3 (OR = 0.77, 95% CI: 0.65–0.91, *p* = 0.002). Trend testing also confirmed a significant negative correlation between DI-GM and diarrhea (*p* = 0.010). When the dietary components were separated into “beneficial score” and “unfavorable score,” we found that each 1-point increase in the beneficial score was associated with further reductions in diarrhea risk (OR = 0.91, 95% CI: 0.87–0.96, *p* < 0.001), whereas the unfavorable components did not show a similarly robust association in this model. The RCS analysis (Figure 2) indicated a significant nonlinear relationship between beneficial score and diarrhea risk (*p* = 0.039). In contrast, the association between the total DI-GM and diarrhea appeared linear (*p* = 0.062), and no notable nonlinear relationship was observed for the unfavorable score (*p* = 0.073).

**Table 2:** Association between DI-GM and diarrhea.

Characteristics	Diarrhea			
	Model 1		Model 2	
	OR (95% CI)	*P*-value	OR (95% CI)	*P*-value
DI-GM	0.9 (0.87 ~ 0.93)	<0.001	0.95 (0.91 ~ 0.98)	0.005
DI-GM group				
0–3	Ref		Ref	
4	1.03 (0.89 ~ 1.19)	0.723	1.08 (0.93 ~ 1.25)	0.324
5	0.90 (0.77 ~ 1.04)	0.154	0.99 (0.85 ~ 1.15)	0.887
≥6	0.62 (0.53 ~ 0.73)	<0.001	0.77 (0.65 ~ 0.91)	0.002
Trend test		<0.001		0.010
Beneficial to gut microbiota	0.87 (0.83 ~ 0.91)	<0.001	0.91 (0.87 ~ 0.96)	<0.001
Unfavorable to gut microbiota	0.97 (0.92 ~ 1.02)	0.194	1.00 (0.94 ~ 1.06)	0.900

DI-GM, dietary index for gut microbiota; PIR, poverty income ratio; BMI, body mass index; CVD, cardiovascular disease; CI, confidence interval; OR, odd ratio.

The Model 1 was adjusted for age and gender, the Model 2 was adjusted for age, gender, race, marital status, education level, PIR, physical activity, smoking status, alcohol intake, BMI, total calories intake, diabetes, CVD, hypertension, hyperlipidemia, and depression.

The DI-GM ranges from 0 to 13 (including beneficial to gut microbiota [ranges from 0 to 9] and unfavorable to gut microbiota [ranges from 0 to 4]) and grouped according to 0–3, 4, 5, and ≥6.

**Figure 3:**
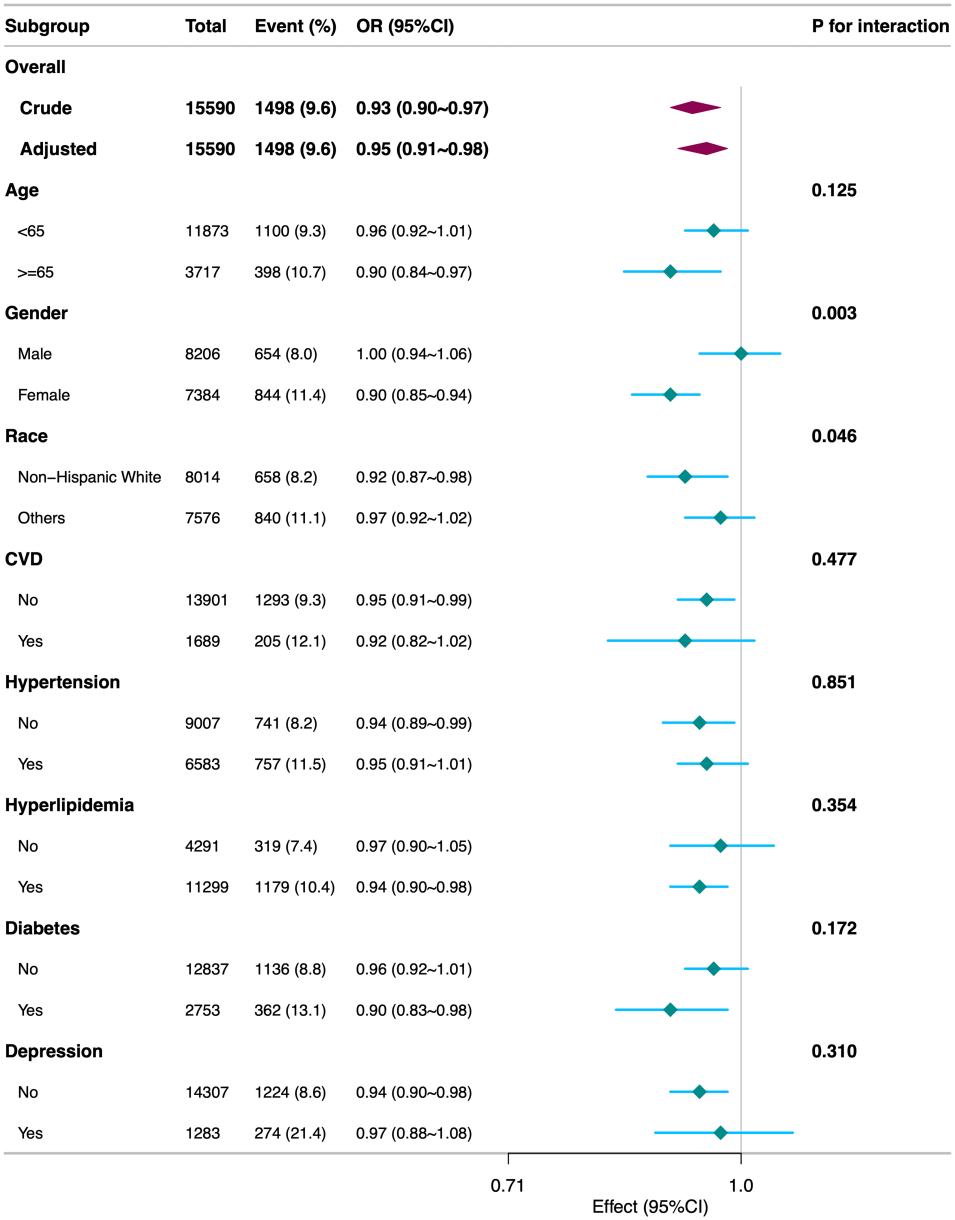
Subgroup analyses of the association between DI-GM and diarrhea among participants. DI-GM, dietary index for gut microbiota; PIR, poverty income ratio; BMI, body mass index; CVD, cardiovascular disease; CI, confidence interval; OR, odd ratio. The model was adjusted for age, gender, race, marital status, education level, PIR, physical activity, smoking status, alcohol intake, BMI, total calories intake, diabetes, CVD, hypertension, hyperlipidemia, and depression.

Stratified analyses by age, gender, race, CVD, hypertension, hyperlipidemia, diabetes, and depression (Figure 3) revealed that the inverse association between DI-GM and diarrhea was generally consistent across most subgroups, underscoring the robustness and potential broad applicability of DI-GM. Notably, significant interactions were observed between DI-GM and gender (*p* = 0.003), as well as between DI-GM and race (*p* = 0.046).

### 3.3 Sensitivity analyses

Multiple imputation for missing data (Supplementary Table 2) confirmed the robustness of the inverse association between DI-GM and diarrhea (adjusted OR = 0.96, 95% CI: 0.93–0.99, *p* = 0.021). Moreover, compared with the 0–3 group, those with DI-GM ≥6 remained at a significantly lower risk of diarrhea (OR = 0.82, 95% CI: 0.71–0.96, *p* = 0.013).

## 4 Discussion

Using large-scale, cross-sectional NHANES data from 2005 to 2010, this study provides the first systematic examination of the association between the newly proposed Dietary Index for Gut Microbiota (DI-GM) and diarrhea. Our key findings include the following: after adjusting for demographic, lifestyle, and health-related confounders, each 1-point increase in DI-GM was associated with a 5% reduction in diarrhea risk, with DI-GM ≥6 conferring a substantially lower risk than DI-GM scores of 0–3. This relationship remained robust after multiple sensitivity analyses. Further breakdown of DI-GM components revealed that “beneficial” factors (e.g., high fiber, fermented dairy, whole grains) exerted the most pronounced effect on decreasing diarrhea risk, while the contribution of “unfavorable” components (e.g., high-fat diet, red meat, processed meat, refined grains) was less pronounced under the current model.

Dietary influence on gut microbiota and its potential role in diarrhea have historically been emphasized. For instance, fermented dairy products (categorized as beneficial in DI-GM) may play a key role in maintaining gut homeostasis and reducing inflammation. A randomized controlled trial showed that a diet rich in fermented foods significantly enhanced gut microbial diversity and reduced inflammatory markers, potentially stabilizing the intestinal mucosal barrier and reducing fluid losses, thereby lowering diarrhea risk (33). Conversely, refined grains, classified as unfavorable in DI-GM, are common in Western diets. High intake of refined grains or sugars can not only raise blood glucose levels but also contribute to gut and neural inflammation (34), ultimately exacerbating intestinal dysfunction and increasing the likelihood of diarrhea.

Initially introduced by Kase et al. (16), DI-GM was designed to assess how overall dietary patterns affect gut microbial diversity. Subsequent work by Huang and Zhang in the NHANES population reported inverse associations between DI-GM and diabetes, as well as constipation (17, 18). Our study complements these findings by focusing on diarrhea, demonstrating that higher DI-GM scores were similarly linked to lower risk of a functional gastrointestinal outcome. Together, these results highlight the importance of comprehensive “diet–microbiota” interactions in modulating disease risk and underline DI-GM’s potential applicability to various functional bowel disorders.

Compared with traditional approaches focusing on individual nutrients (e.g., probiotics, prebiotics) or anti-diarrheal medications, DI-GM synthesizes multiple gut microbiota-related dietary components and offers a more holistic measure of overall diet quality. Our results suggest that greater emphasis on beneficial components (such as fermented dairy products, whole grains, and fiber) may critically lower diarrhea risk. Clinicians and dietitians could thus consider incorporating DI-GM or similar metrics into dietary guidance to help patients optimize their gut microbiota. For those suffering from chronic diarrhea, IBS, or other functional bowel disorders, a “microbiota-friendly” dietary intervention could alleviate symptoms, prevent recurrences, and facilitate weight management (35). From a public health perspective, promoting microbiota-oriented dietary patterns may serve as an economical and feasible strategy for diarrhea prevention and management on a broader scale.

Several mechanisms may underlie DI-GM’s beneficial effect on diarrhea risk. First, high fiber, fermented dairy products, and other “microbiota-friendly” foods provide fermentation substrates for beneficial microbes, stimulating short-chain fatty acid (SCFA) production (e.g., butyrate, propionate, and acetate) (36, 37). These metabolites not only supply energy to epithelial cells but also enhance the expression of tight junction proteins (e.g., ZO-1, occludin), strengthening the intestinal barrier and reducing fluid loss (38). In contrast, diets high in refined carbohydrates and fats may diminish SCFA production, compromise barrier integrity, and increase diarrhea risk (39, 40). Next, dietary patterns heavily shape microbial diversity and the relative abundance of beneficial taxa. A more diverse, prebiotic-rich microbiota can suppress colonization and overgrowth of pathogenic or opportunistic bacteria (41). High-fat, high-sugar diets, however, often reduce beneficial microbes and disturb microbial ecology, thereby increasing susceptibility to diarrhea (42). Additionally, excessive intake of fat and red meat alters bile acid secretion and metabolism. An imbalanced microbiota may be less capable of effectively transforming and binding excess bile acids in the colon, promoting mucosal irritation and fluid secretion, which precipitate diarrhea (43). Conversely, probiotics or certain fibers can mitigate these processes and, by regulating enterochromaffin cell-derived serotonin (5-HT), maintain normal intestinal peristalsis (44). Finally, beyond local physiological effects, dietary-induced microbial changes may also modulate central nervous system function via neurotransmitter production (e.g., 5-HT, GABA), immune signaling, and vagal pathways (45). Functional diarrhea often correlates with emotional stress, anxiety, or depression (46). A healthy, diverse microbiota may help modulate central stress responses and improve tolerance to gastrointestinal perturbations, thus reducing diarrhea incidence or severity.

Despite these promising findings, several limitations warrant attention. First, the study used cross-sectional data, which precludes causal inference and does not rule out reverse causation (i.e., diarrhea may alter dietary patterns). Second, we relied on NHANES data from 2005 to 2010, the only period during which the Bowel Health Questionnaire provided consistent Bristol Stool Form Scale-based definitions of diarrhea; therefore, changes in dietary patterns or lifestyle factors in more recent years are not captured. Third, medication use was not considered, potentially overlooking key interactions between pharmacological interventions, dietary habits, and bowel function. Fourth, we did not account for certain underlying gastrointestinal disorders, including exocrine pancreatic insufficiency, which may confound gut microbiota composition and influence diarrhea risk (47). Fifth, the definition of diarrhea was based on self-reported data, which may introduce subjective bias and omit details about duration and frequency, leading to possible misclassification. Sixth, NHANES depends on 24 h dietary recalls, which can introduce recall bias and may not fully reflect long term dietary habits. Seventh, the absence of direct microbial sequencing and inflammatory or immune biomarker assessments limited our ability to identify specific microbial taxa or metabolic pathways that might mediate the observed relationship between diet and diarrhea risk. Finally, only participants who completed the Bowel Health Questionnaire were included, which may introduce selection bias and affect the generalizability of our findings.

From a translational perspective, our findings underscore the potential benefit of a gut-microbiota-friendly diet, as indicated by higher DI-GM scores, in preventing or mitigating diarrhea. Clinically, the DI-GM framework may assist in guiding dietary counseling for individuals with functional bowel disorders, where targeted dietary modifications could alleviate symptoms and reduce recurrence. On a public health level, emphasizing key beneficial components (e.g., high-fiber foods, fermented dairy) may serve as an accessible, cost-effective strategy to improve gut health and lower the burden of gastrointestinal symptoms. Further research in prospective cohorts or randomized controlled trials, ideally integrating multi-omics approaches (e.g., metagenomics, metabolomics) along with immune and gut barrier assessments, is needed to confirm causality and refine personalized dietary interventions for diarrhea and other gut-microbiota-related conditions.

## 5 Conclusion

In summary, this cross-sectional study of a large NHANES sample reveals that higher DI-GM scores are significantly associated with lower diarrhea risk. Future research in multi-center prospective cohorts and intervention trials using multi-omics techniques may further clarify the causal pathways linking DI-GM to intestinal health and offer more precise guidance for individualized nutritional intervention and public health strategies to prevent and manage diarrhea.

## Data Availability

The datasets presented in this study can be found in online repositories. The names of the repository/repositories and accession number(s) can be found at: Publicly available datasets were analyzed in this study. All data entered into the analysis were from NHANES, which is publicly accessible to all.

## Footnotes

^1^ https://www.cdc.gov/nchs/nhanes/index.html
