# Supplementary material for: Association between dietary index for gut microbiota and diarrhea among US adults: a cross-sectional analysis of NHANES 2005–2010
Source: Front Nutr. 2025 Apr 11;12:1566314. doi: 10.3389/fnut.2025.1566314 (PMC12021626; doi:10.3389/fnut.2025.1566314)
Supplement: Supplementary file 1 [file Table_1.docx]

**Supplementary Materials Files**

To: **Association between a Newly Proposed Dietary Index for Gut Microbiota and Diarrhea: A Cross-Sectional Study Based on NHANES**

by Xiaoqiang Liu, Xiaobo Liu, Yingxuan Huang, Chanchan Lin, Xinqi Chen, Yingyi Li, Yisen Huang, Yubin Wang

**Supplementary Table 1** **Components and scoring criteria of DI-GM in NHANES.**

| **Components of**  **DI-GM** | **Food items included in NHANES** | **Scoring criteria** |
| --- | --- | --- |
| **Beneficial to gut microbiota** | Avocados, Broccoli, Chickpeas, Coffee, Cranberries, Fermented dairy (including yogurt, cheese, kefir, sour cream, buttermilk), Fiber, Soybean (including Soy milk, Tofu), Whole grains | Score 1 - Consumption ≥ sex-specific median  Score 0 - Otherwise |
| **Unfavorable to gut microbiota** | Refined grains, Processed meat, Red meat | Score 0 - Consumption ≥ sex-specific median  Score 1 - Otherwise |
|  | High-fat diet (% energy) | Score 0 - Consumption ≥ 40%  Score 1 - Otherwise |

Abbreviations: DI-GM, dietary index for gut microbiota; NHANES, National Health and Nutrition Examination Survey.

**Supplementary Table 2 Association between DI-GM and diarrhea after** **multiple imputation**

| **Characteristics** | **Diarrhea** | | | | |
| --- | --- | --- | --- | --- | --- |
|  | **Model 1** | |  | **Model 2** | |
|  | **OR (95% CI)** | ***P* -value** |  | **OR (95% CI)** | ***P* -value** |
| DI-GM | 0.9 (0.87~0.93) | <0.001 |  | 0.96 (0.93~0.99) | 0.021 |
| DI-GM group |  |  |  |  |  |
| 0-3 | Ref |  |  | Ref |  |
| 4 | 1.03 (0.89~1.19) | 0.723 |  | 1.07 (0.93~1.23) | 0.348 |
| 5 | 0.90 (0.77~1.04) | 0.154 |  | 1.02 (0.88~1.17) | 0.805 |
| ≥6 | 0.62 (0.53~0.73) | <0.001 |  | 0.82 (0.71~0.96) | 0.013 |
| Trend test |  | <0.001 |  |  | 0.048 |
| Beneficial to gut microbiota | 0.87 (0.83~0.91) | <0.001 |  | 0.93 (0.89~0.97) | 0.001 |
| Unfavorable to gut microbiota | 0.97 (0.92~1.02) | 0.194 |  | 1.01 (0.95~1.07) | 0.761 |

Abbreviations: DI-GM, dietary index for gut microbiota; PIR, poverty income ratio; BMI, Body Mass Index; CVD, cardiovascular disease; CI, Confidence interval; OR, Odd Ratio.

The Model 1 was adjusted for age and gender, the Model 2 was adjusted for age, gender, race, marital status, education level, PIR, physical activity, smoking status, alcohol intake, BMI, total calories intake, diabetes, CVD, hypertension, hyperlipidemia, and depression.

The DI-GM ranges from 0–13 (including beneficial to gut microbiota [ranges from 0–9] and unfavorable to gut microbiota [ranges from 0–4]) and grouped according to 0–3, 4, 5, and ≥ 6.
